# Barriers and facilitators to integration of screening for hypertension, diabetes mellitus and dyslipidaemia, among adult people living with HIV at district hospital ART clinics in Southern Malawi

**DOI:** 10.21203/rs.3.rs-5373585/v1

**Published:** 2024-12-09

**Authors:** Kondwani G.H. Katundu, Victoria Mukhula, Zaithwa Matemvu, Angel J. Mtonga, Myness Kasanda-Ndambo, Adriano F. Lubanga, Monalisa G. Malenje, Wongani Nyangulu, Grace Momba, Isotta Triulzi, Noel Kalanga, Mwapatsa Mipando, Alinane Linda Nyondo-Mipando, Mina C. Hosseinipour

**Affiliations:** Kamuzu University of Health Sciences; Malawi-Liverpool-Wellcome Trust Clinical Research Programme; Kamuzu Central Hospital; Kamuzu University of Health Sciences; Kamuzu University of Health Sciences; Kamuzu University of Health Sciences; Zomba Central Hospital; Kamuzu University of Health Sciences; Chikwawa District Hospital; Scuola Superiore Sant’Anna; Kamuzu University of Health Sciences; Kamuzu University of Health Sciences; Kamuzu University of Health Sciences; University of North Carolina projects

**Keywords:** Integration, Atherosclerotic cardiovascular diseases, Sub-Saharan Africa, people living with HIV, hypertension, DM mellitus, dyslipidaemia, Malawi, implementation strategies

## Abstract

**Background:**

Atherosclerotic cardiovascular diseases (ASCDs) are a significant health concern globally and in Sub-Saharan Africa (SSA), particularly for people living with HIV (PLWH). Hypertension, diabetes mellitus (DM), and dyslipidaemia significantly increase the risk of ASCDs, and integrating screening for these conditions in public health facilities remains challenging in Malawi. This study aimed to explore the barriers and facilitators to integrating screening for hypertension, DM and dyslipidaemia among adult PLWH at district hospital ART clinics in Southern Malawi.

**Methods:**

This was mixed-methods study conducted between November 2021 and April 2022. Quantitative data was collected from retrospective ART clinic records from between 2016 and 2020 (n = 875) from five district hospital ART clinics and informed the subsequent qualitative data collection, guided by the Consolidated Framework for Implementation Research (CFIR) in three purposively selected district hospital ART clinics. The qualitative aspect included in-depth interviews, focus group discussions, and key informant interviews. Non-participant observations were also conducted to assess the availability of functional screening equipment. Descriptive statistics were used to analyse the quantitative data while the qualitative data was analysed using thematic analysis.

**Results:**

One district hospital ART clinic facility only performed the screening for hypertension and DM (40% and 9.84% at the commencement of ART and 39.4% and 5.14% in 2021). Facilitators for integration included time efficiency, patient information integration, existing infrastructure utilization, organisational incentives and training. Barriers included clinic ow delays, additional strain on a limited workforce, lack of prioritization and resources, increased workload and inadequate knowledge. PLWH expressed fear of screening and diagnosis without available medication.

**Conclusion:**

The study found poor integration of hypertension, DM and dyslipidaemia screening among PLWH in Southern Malawi, but highlighted opportunities for successful implementation. Our study emphasizes the feasibility of the intervention and the importance of coordination between HIV and NCD care services in low-income settings such as Malawi.

## BACKGROUND

Atherosclerotic cardiovascular diseases (ASCDs), mainly ischaemic heart disease and stroke, are the leading causes of morbidity and mortality globally and in Sub-Saharan Africa (SSA) [1, 2]. Hypertension, diabetes mellitus (DM) and dyslipidaemia are major risk factors for ASCDs [1, 3]. People living with HIV (PLWH) have an increased risk of ASCDs, and the presence of hypertension, dyslipidaemia or DM increases the risk of ASCD up to 2.4-fold in PLWH [4–11]. Malawi has one of the world’s highest burdens of ASCDs in PLWH, where between 131.62 and 1765.11 attributable disability-adjusted life years (DALYs) per 100,000 PLWH are associated with ASCDs [12]. Undiagnosed and poorly managed hypertension, DM and dyslipidaemia are the major contributing factors to the burden of ASCDs in Malawi, and disproportionately affect PLWH due to their increased risk for ASCDs [13]. Strategies towards screening and managing these risk conditions are imperative to reducing the morbidity and mortality associated with ASCDs in PLWH.

The World Health Organization and the International Association of Providers of AIDs Care recommend screening PLWH for DM, dyslipidaemia, and hypertension at initiation of antiretroviral therapy (ART), and at least annually after that [14–16]. Similarly, the most recent Malawi National Clinical Guidelines for the Management of HIV recommend screening people living with HIV (PLWH) who are 40 years and older for hypertension and DM at the initiation of ART and annually after that [17]. However, the guidelines do not include screening for dyslipidaemia, a significant risk factor for ASCVDs in PLWH [17]. Despite these recommendations and guidelines, many public health facilities in Malawi are lagging in integrating hypertension, DM, and dyslipidaemia screening and management among PLWH [18–20]. Accordingly, there is a need to increase the uptake of integration of screening and managing these conditions in public health facilities, to reduce the burden of ASCDs among PLWH in Malawi.

An integrated approach to screening and managing ASCD risk conditions in PLWH in low to middle-income countries, including in SSA has been proven feasible. However, barriers to implementing this integrated care include limited staffing, space, supplies, and screening tools [21, 22]. However, in Malawi, there is limited data on the barriers and facilitators to integrating screening for hypertension, DM, and dyslipidaemia in PLWH. Understanding these barriers and facilitators in the Malawian setting is critical to developing effective and contextualised implementation strategies for screening and managing DM, hypertension, and dyslipidaemia among PLWH in public health facilities.

This study aimed to determine the rates and factors that influence the integration of screening of hypertension, DM and dyslipidaemia among adult people living with HIV at district hospital ART clinics in Southern Malawi.

## METHODS

### Study design.

**Figure 1:**
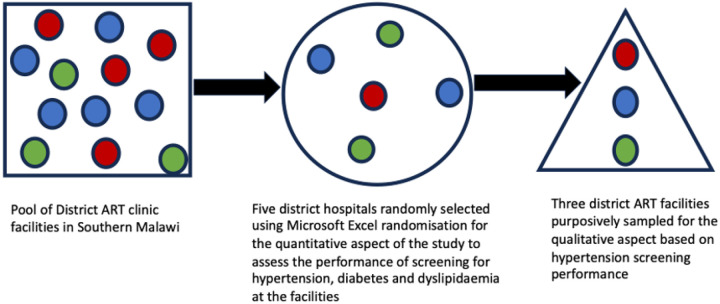
A schematic diagram of the sequential explanatory mixed methods design of the study. Quantitative data was collected from five randomly selected facilities and informed the collection and analysis of the subsequent qualitative aspect of the study from three purposefully selected health facilities.

**Figure 2:**
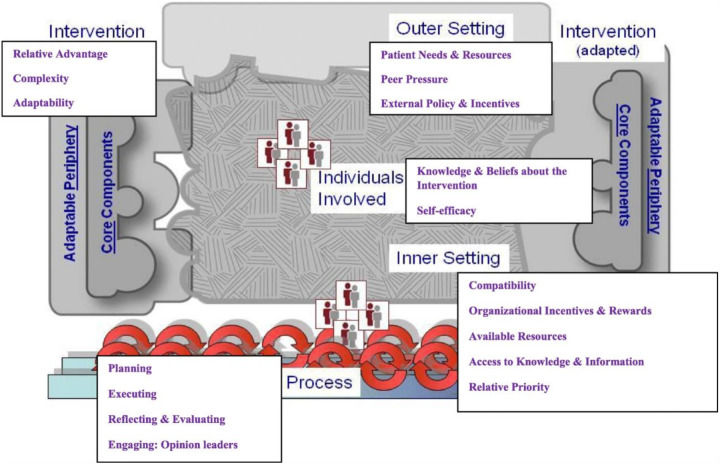
A depiction of the consolidated framework for implementation research (CFIR), indicating the five domains and its constructs [25]. three of the five domains of the CFIR, namely, the intervention, the inner setting and the characteristics of the individual were investigated.

This was a mixed-methods study using a sequential explanatory design, which was conducted between November 2021 and April 2022 (Fig. 1). The sequential explanatory mixed methods design aims to collect and analyse quantitative data, which then informs the collection and analysis of qualitative data [23, 24]. Quantitative data was collected from retrospective ART clinic records of between 2016 and 2020, of PLWH aged 40 years and above on the performance of screening of hypertension, DM and dyslipidaemia at the commencement of ART, and within the past 12 months from the study date. The qualitative data collection was guided by the consolidated framework for implementation research (CFIR), where three of the five domains of the CFIR, namely, the intervention, the inner setting and the characteristics of the individual were investigated (Fig. 2) [25]. In addition, non-participant observations were done, and we observed and recorded the availability and functionality of screening tools for hypertension, DM, and dyslipidaemia at the ART clinics.

### Study setting

The study was conducted in five District Hospital ART clinics in the Southern Region of Malawi namely: Machinga, Mangochi, Neno, Chiradzulu and Thyolo. The public healthcare system in Malawi comprises four tertiary care hospitals at a regional level, twenty-nine district hospitals which provide a mix of primary and secondary care spread across each district, and community health centres that provide primary health care in communities. There are 12 district hospital ART clinics in the Southern Region of Malawi. Each district has a District Health Management Team (DHMT), responsible for ensuring healthcare service delivery in the district hospital and community health centres. Each district hospital has a designated outpatient ART clinic where PLWH are screened and treated for HIV-related comorbidities following the national guidelines, at no cost. Clinicians, nurses, and counsellors staff the ART clinics. The district hospitals were selected as suitable centres for the study due to the existence of ready structures for HIV/NCD integrated care. All the five district hospitals are supported by the government of Malawi. However, Neno district hospital receives additional support from Partners in Health (PIH). PIH generally supports Neno district hospital regarding clinical human resource, laboratory facilities and procurement of essential medication. Specifically, In partnership with PIH, Neno district has longitudinal integrated chronic care clinics (IC3) caring for HIV and chronic NCDs [26].

### Sampling and sample size

#### Quantitative retrospective audit sampling and sample size

For the quantitative aspect, five district hospital ART clinics Machinga, Mangochi, Neno, Chiradzulu and Thyolo were randomly selected from a list of all 12 district hospital ART clinics in the Southern region using Microsoft Excel random selection function. In each of the selected district ART clinics, we systematically selected 175 participant files by reviewing every other le of adult PLWH aged at least 40 years attending the ART clinic in each district hospital (total 875) who commenced ART between the years 2016 and 2020. Records older than 2016 were excluded because screening for hypertension at ART initiation and yearly after was introduced in the Malawi National HIV guidelines in 2016 [27]. The sample size calculation was based on detecting at least 10% difference between the highest and lowest performance on hypertension screening among the 5 district hospitals at a power of 80% and alpha of 0.05. We also excluded all les of PLWH who had prior diagnosis or were on treatment for hypertension, DM or dyslipidaemia before ART initiation; and people who had initiated ART elsewhere other than the ART clinic at the district of study.

#### Qualitative sampling and sample size

We purposely selected three out of the five district hospital ART clinics for the qualitative study, based on their performance in the screening for hypertension employing a maximum variation approach so that we draw from facilities with varying experiences and performance [28]. We intended to select one district hospital ART clinic rated as the highest, intermediate, and lowest performing ART clinic based on hypertension screening rating at the initiation of ART, respectively from the quantitative results of this study. The performance of hypertension screening at ART initiation expressed as a proportion of PLWH screened of the total reviewed les at each district hospital ART clinic was used to rate the five clinics from the highest to the lowest. However, one ART clinic performed as the highest and the other four performed equally as the lowest performing clinics. In that case, we randomly selected two facilities from the four using Microsoft Excel randomization function. Ultimately, Neno (NN), Chiradzulu (CZ) and Mangochi (MC) district ART clinics were selected for the qualitative study.

For the participants of the qualitative study, a purposive approach with maximum variation was utilised and it included participants of varying characteristics to widen the scope of responses [29]. The sampling frame and sample size is illustrated in Table 1.

### Data Collection

#### Quantitative retrospective audit

A data extraction form was used to collect data on the screening of hypertension, DM, and dyslipidaemia at ART initiation and the previous 12 months from the study date. The data was extracted from the ART clinic les of PLWH aged at least 40 years at each of the five district ART clinics who initiated ART from 2016 and 2020. The data was de-identified and entered into an excel sheet by three trained research assistants. However, we still collected data on the screening for DM and dyslipidaemia since these are significant risk factors for ASCDs, even though dyslipidaemia was not yet included in the Malawi HIV Clinical Management guidelines. The data extraction form was reviewed by experts to ascertain its validity.

#### Qualitative aspect

We collected qualitative data using in-depth interviews (IDIs) with individuals of different characteristics in terms of sex, marital status, body mass index and having any known cardiovascular risk factor with PLWH to allow for maximum variation. The IDIs were conducted in Chichewa which is the local language. We also conducted focus group discussions (FGDs) with the healthcare providers at the ART clinics. Key informant interviews (KII) were conducted with members of the DHMT at the district hospitals. Both the FGDs and the KII were conducted in English and participants were also allowed to contribute their opinion in Chichewa if they felt so. All interviews were audio recorded.

We used semi-structured interview guides for the IDIs, FGDs, and KIIs based on the three domains of CFIR of interest (see Table 2). A supplementary le has been provided containing the interview guides in English, which were used for the qualitative data collection (Supplementary File 1). All interviews were facilitated by three trained research assistants, who were conversant with qualitative research using the guides which were pre-tested at Chiradzulu District Hospital before use, and the results were used to re ne the guides. The IDIs, KIIs, and FGDs were conducted in private environments as selected by the participants. After each interview, the research assistant summarised the interview findings and shared with the participant to confirm, as a form of member checking. Saturation was realised when there were no more new ideas and 2 more interviews were added to ascertain the saturation.

#### Quality of the qualitative data

To ensure the dependability of our qualitative data, we included participant quotes as an indication that the results were founded on the participants’ statements. Additionally, we ensured credibility of our findings by using multiple approaches to our data collection such as FGDs, IDI and KII with healthcare workers, PLWH, and members of the DHMT at each of the health facilities where the qualitative study occurred. Moreover, to adequately address reflexivity, the research team often reflected on their experiences and knowledge of ART and NCD clinical care services to avoid their experiences from guiding the data collection and influencing the data analysis. The research team often met and shared their written experiences, assumptions and knowledge of ART and NCDs care during the data collection and analysis phase as a measure of addressing reflexivity [30–31].

### Non-participant observations

We observed the ART clinic internal environment for the presence of blood pressure measuring machines and whether they were or not functional. We also observed the presence of a glucometer, and HBA1C portable machine or a laboratory station within the ART clinic. Additionally, we visited the hospital laboratories and checked the availability of DM and dyslipidaemia screening materials and whether the laboratories receive orders for DM and dyslipidaemia orders from the ART clinic. The observations were guided by a checklist form. All findings were recorded and collated to complement the other findings.

### Data Management and Analysis

The records for the research participants were de-identified at the data collection point. Only the investigators had access to the identification key. Hard copy records were stored in a locked filing cabinet at the Kamuzu University of Health Sciences (KUHeS). The de-identified records were stored on a password-protected computer only accessible to the investigators. Quantitative data were entered in Microsoft Excel spreadsheets. Statistical analysis was done using Stata17 (Stata Corp, USA) software. Descriptive statistics were expressed as means or medians for continuous data such as participant age, and proportions for categorical data such as proportion of PLWH screened for hypertension, DM and dyslipidaemia. To compare categorical data, the Chi-square was used for independent variables. The t-test was used for hypothesis testing, to analyse the difference in mean difference. In all cases, a p-value of less than or equal to 0.05 was considered statistically significant.

Transcripts were transcribed verbatim in Chichewa, then translated to English, and later imported into NVivo 12 for data management. The transcripts were anonymised, and each participant was assigned a participant identity (ID) number. The first author (KGHK), who listened to all audio recordings and confirmed the translations from Chichewa to English, double-checked the transcription and translation work completed by MGM. The data were analysed using a thematic approach. KGHK and MKN familiarised with the data set through immersion in the repetitive and active reading of transcripts [32]. To guarantee consistency and dependability in coding, KGHK and MKN separately went over the first three transcripts line by line and used deductive reasoning to draw conclusions about related ideas that kept coming up in the data [33] with reference to the CFIR [27]. The first codebook was generated from the first three transcripts through a consensus process by looking at commonalities and differences until a final codebook was created [32]. All authors agreed on the final codebook and discussed areas of divergence. The data coder was oriented to the codebook and held iterative meetings with the researchers to discuss the inclusion of more codes in the codebook or merging overlapping codes. MKN then transferred all the English-language transcripts into NVivo 12 for data management and applied the codebook to all transcripts. The MKN, LNM, and KGHK identified relationships between these codes; repeatedly identified codes were merged; and themes and sub-themes were generated from these codes. We chose participant quotes for each theme and sub-theme, summarizing the main points [33]. For each theme and sub-theme, we selected participant quotes that encapsulated the key ideas [33]. The data were organized under the predetermined themes that were deduced from the CFIR framework. Each theme was examined in comparison to the audio and transcripts as a measure of checking that themes were supported and aligned with appropriate quotes. Themes lacking supporting data were eliminated, and those with comparable data were merged.

## RESULTS

### Quantitative Results

We reviewed a total of 875 randomly selected records of PLWH aged 40 years and older attending the ART clinics at Machinga, Mangochi, Neno, Thyolo and Chiradzulu district (175 records per district). Table 2 below shows the characteristics of the participants.

There were more records of male PLWH (55%) reviewed in the study than females. The participants had a median age of 49 years and had been on ART for 41 months. At the commencement of ART, approximately 60% of the PLWH were on a tenofovir/lamivudine/efavirenz combination of ART. In contrast, at the time of the study, 98% were on a tenofovir/lamivudine/dolutegravir ART regimen. At the time of the study, compared to the time of commencement on ART, the rate of overweight increased significantly.

We compared the facilitators and barriers between the highest performing facility and the two lowest performing facilities on the integration of screening of the three NCDs at the ART clinics based on the three CFIR domains; the intervention, the inner setting and the individual characteristics and their sub themes based on the analysis. However, this categorisation is not rigid, as some of the subthemes are interrelated and were categorized as such for logical descriptive purposes.

### Facilitators and barriers related to the intervention

The facilitators related to the intervention included perceived relative advantage and cost-effectiveness, that the integration would not be complex and the willingness of the HCW to provide a chance for trial. The barriers were related to the perceived increase in the workload and paperwork, and waiting time associated with the integration of the screening for the NCDs at the ART clinics (Table 4).

### Perceived relative advantage and cost-effectiveness.

At both the high (NN) and low performing sites (CZ and MC), PLWH and HCWs expressed that the integration of screening for hypertension, DM and dyslipidaemia would provide care at a one-stop centre, and that this would be cost-effective. This was perceived as an important enabler.

“I have seen that the benefits are there because of this integration, since it’s like we are killing two birds with one stone. Instead of coming here frequently and work being halted back home we come here only once and get all the services and it means that the rest of the remaining days, I will be home doing other things instead of visiting the hospital more often…” NN, female IDI.

Additionally, the integration was perceived as an initiative which saves time and transport costs for the PLWH accessing the care for both HIV and the NCDs. HCW at NN also reported that the integration improves patient retention in care, since all services are provided at one appointment, and reduces overcrowding due to multiple visits. Collectively, PLWH, HCW and the DHMT members at all the facilities stated that the integrated care would provide a complete patient pro le, optimize pharmacotherapy, and promote efficiency in the provision of care.

‘…to my part, I think there will be a proper following of our clients and it will help us understand where the problem is coming from. It will help bring order because the patient will know what to expect when they come to the clinic and linkage will be better. If we are screening them at least once a year, it’s easy to trace them. If the point of care is at one place, it’s easy to follow up. For that same patient to be attending the ART clinic here, the diabetes clinic somewhere else, its hectic for the patients, so it will be helpful that the client gets all the services at one point of care.’ CZ, FGD participant, Female Nurse.

### Complexity and trialability

At NN, implementation of the intervention was reported to be reasonably less-complex in conjunction with other NCDs and ART related services. Similarly, HCW at CZ and MC indicated that integration of care was seen as a feasible endeavour, as there are already existing structures such as the clinic rooms which can accommodate the intervention.

‘.… okay, for me the tra c ow will be ne because we already have space there where they do the registration, and we already have the human resource who do the vital signs. So as they are doing the registration, the weighing so the blood pressure checks can be done there. For the lab tests, all we need is proper planning, but we can handle it the way we do at the routine diabetic clinic. And yes, we are willing to give the intervention a trial’ CZ, KII Female DMHT member.

### Clinic flow delays and costs

HCWs at CZ and MC stated that integrating the services may create delays in clinic waiting times, as it would take longer to review each patient more thoroughly.

‘Clients will be delayed because of the process which is too long to finish and there will be long period of waiting for the clients to access these services’ MC, FGD male clinician.

In addition, HCW in CZ and MC mentioned that system-establishment and standard operating procedures (SOPs), which would be required to fully implement the intervention would be expensive to set up.

‘To implement this initiative will be expensive because we will need to develop clear SOPs in liaison with all the departments, the lab team and the ART clinical and nursing staff. It is doable but we need to involve everyone in the planning phase.’ CZ, KII, female DHMT member.

Another generally reported concern with integrating NCD screening at the ART clinics was the possibility of increased paperwork for the HCWs as there would be more documentation for each person receiving the services. Along with this increased workload, there were concerns about integrating the data flow.

‘The number one challenge is that there is a lot of paperwork since we haven’t reached a stage where we can use electronic devices when gathering data or information. So, for example, some clients can have three conditions whereby the same PLWH would have diabetes and Asthma for example. This means that he/she has three master cards plus health passport, which means that you have to transfer all notes into them’ NN, FGD, male clinician.

### Compatibility

Another facilitator, especially at NN, was the presence of already existing structures that they could build on, and the partnerships between the hospital and a non-governmental organization which support NCDs care.

‘Okay, the integration that is happening here, we are using the old structures for the ART clinic. The government supports the clinics in terms of medication, and on the other hand other medications are provided only by our partner NGO, especially those medications which the government does not supply.’ NN KI, male DHMT member.

At CZ and MC, inadequate staffing was identified as a major problem, and some HCWs felt that integration would strain the already burdened workforce.

‘If it is going to be implemented now, then the intervention would be an inconvenience to usual service delivery. The integration is important, but a lot of other services are also done here and even though it’s important, there is a need to critically consider its implementation. I don’t know how others have done it, but we need to look at the number of staff who will be able to handle this, are they enough? Because generally there are already a lot of services which we offer here at the ART clinic, and we don’t have enough staff, hence having a lot of pressure in the process. So, we need to think in terms of the ow and where can this fit in and is it going to work if we are to add it there?’ MC, FGD, female nurse.

Nonetheless, it was stated that with an organised clinic ow, the integration would be compatible at facilities such as CZ as reported by the DHMT member at the facility.

‘We have five consultation rooms with one nurse, the support staff and one clinician. We are currently using 3 rooms and the other 2 are not in use. If we devise a good system, we can have a general clinician in one room and then another clinician who is handling the NCDS in the other room and the client ow can be well handled. The patient will go to the nurse if there are no complications and refill medication and off, they go. But if there is a complication, the nurse will see if the patient needs to see the general clinician or the NCD clinician or they can work together in one room for the sake of mentorship, but I think we have space, the ow can be managed very well. CZ, KII, Female DHMT member.

### Facilitators and barriers at the level of the Inner setting

At the inner setting, reported facilitators and barriers pertained to availability of resources, availability of human resource in terms of HCW and motivation for the HCW.

### Available resources for screening and management were a facilitator while lack was a barrier

The HCWs at NN reflected on how the availability of resources such as functioning blood pressure cuffs facilitated the screening process for hypertension. Furthermore, the availability of medicines and other resources such as WiFi also positively impacted work ow and facilitated the implementation of the integration.

‘What motivates me is the availability of medicines. It’s really discouraging to know what to do but have nothing to offer. So, when we have the medicine, then that’s what motivates us. Also, we have very good WiFi, for example, if I want to search for some information to help someone, I will go and check quickly and that also motivates some of us and if you really want to study something, you can easily go there and find information and help our clients. In short, this is the backbone of the hospital, if the clinic fails due to lack of resources, the whole hospital has failed including the top management, so it is the priority’ NN, FGD male clinician.

Nonetheless, at CZ and MC, participants cited a lack of adequate human and screening resources for NCDs, and the lack of NCDs being appropriately prioritized by the government health facilities as limiting factors to the uptake of this integrated approach in the district’s clinics.

‘One of the challenges that is there in integrating the tool is about the human resource because mostly the government, the capacity building is that of the ART component, but as for the NCD is something which is a little bit new especially to the government employees. It’s a component which is very new, and it is not well established in most of the centres but here is something which is now established’ MC, KI, male DHMT member.

Contrary to CZ and MC, NN had dedicated human resource working at the integrated chronic disease facility, supported by their NGO partner institution.

‘A good percentage of the workforce is supported by our NGO partner right now, the good percentage is for the partners, because from the government side we have the shortfall in terms of the clinicians. So, mostly the clinicians are based in the wards, while the the NGO partner clinicians are in the integrated clinic and that is what is happening’.

### NN, KII, male DHMT member

The availability of the human resource at NN facilitated the implementation of the intervention to the satisfaction of PLWH accessing the services.

‘When you have arrived here, they assist you so quickly and it doesn’t take long before leaving for home. The workers are enough in all the rooms and you would enter into the room and you will be screened for BP and you start with checking your weight, then you go for hypertension and then diabetes, then you will go to the nurse and the nurse will show you where to get the drugs and you receive the drugs and then off you go back home. So, this building has so many rooms because the rooms are demarcated properly and once you have arrived here in this building, be assured that you will be assisted and deal with everything right here at once’. NN, IDI, female participant.

In addition to human resources, not having some screening tools was another barrier. This especially applied to the screening for dyslipidaemia, as virtually all the clinics did not typically have the necessary reagents to conduct lipid profiles.

‘If we had enough resources in terms of running the lipids pro les, that could have helped a lot because that is the component which is missing at the moment, but for the hypertension and diabetes we have enough resources because they are the same resources that we usually use in the wards like the BP cuffs, the weighing scales, you talk of glucose test strips and the glucometers.’ NN, KII, male DHMT member.

The HCWs at CZ and MC were also willing to implement the integrated care system, provided their concerns related to availability of resources were addressed.

‘Okay, I think It is a good intervention, I don’t see any challenges to that, especially if we bring in extra resources especially for the biochemical aspect, we may need support for the reagents to be able to screen’ MC, KII, male DHMT member.

At all the health facilities, HCWs highlighted the fact that they rely on donor support for screening equipment, and the hospital itself may not always have this equipment in stock provided by the government.

‘……They are screened right here, an NGO donated a blood pressure machine about 2 months ago and the screening is done right here’ CZ, FGD, female nurse.

### Organisational incentives and access to training were facilitators

At NN, the HCWs stated that the integrated clinic is a priority for their district hospital and in conjunction with their NGO partner, efforts are made to have continuous professional development training. This was expressed as a facilitator for the implementation of the intervention.

‘…and I can remember last year there were ART trainings and our NGO partner supported some of us to go for the training under the organization nances and not as the ministry of health.’ NN, FGD, female nurse

At CZ and MC, many of the HCWs involved in routine care lamented the lack of access to trainings and refresher courses and being left out for such activities.

‘What happens most times is that seniors will go to the training and then they will just come to tell you to start doing certain things without explaining why we are doing the things, so it gets so hard to explain to clients why we are doing some things’ CZ FGD

The HCW at CZ and MC also believed that NCDs among PLWH were not a priority at their facilities.

‘…no, currently our priority is to screen for the chronic cough for suspected TB, but not the NCDs. But as I said when you go to the wards today you will not see many PLWH admitted with infections but the NCDs, and these people have been coming every 3 months to the ART clinic, but they were never checked’ CZ, KII female DHMT member.

### Facilitators and barriers pertaining to the individual characteristics level

At the individual level, the presence or absence of knowledge, positive attitude and beliefs about the intervention for both the PLWH and the HCW, assurance of availability of treatment once diagnosed by PLWH, and self-efficacy for managing NCDs by HCW were described as either facilitators or barriers for the implementation of the integrated screening for the NCDs at the ART clinics.

### Knowledge and beliefs about the intervention

At all the clinics, HCW and PLWH had some knowledge on the link between NCDs and HIV infection. Furthermore, they acknowledged that screening for these conditions is important for early recognition and management and lifestyle modifications.

‘Of course, individuals who are on ART can also have NCDs like hypertension and diabetes. There are some ART regimen drugs that pose a risk for diabetes. We have observed that there are some people who have developed diabetes whilst on ART ………’ NN, KII, male DHMT member.

PLWH recognised that screening for NCDs would give them the opportunity to either be treated early, or live assured that they are not sick.

‘..if screening is done, we will live a fear free life because one can be given medical management if he or she is found to have one of these conditions, this can either be instructions on how to live better or medications.’ MC, IDI, female participant

uncertainty of availability of medication once has been diagnosed of either hypertension or diabetes was expressed as a demotivating factor, and hence a barrier. PLWH at CZ and MC feared that the health facilities may not be able to treat them for the conditions after the screening process.

‘My only worry will be on the treatment whereby you have been tested and diagnosed with the disease and yet the treatment is not there, that will be my main concern on the whole process.’ CZ, IDI male participant.

Additionally, lack of accurate information regarding hypertension, diabetes and dyslipidaemia was a barrier toward motivation to undergo screening among PLWH. Some PLWH were afraid of receiving a diagnosis of blood pressure, as they perceived it to be fatal and would not want to know their status.

‘Rumours about these conditions are scary, some say when one has high blood pressure, he or she will die or if one compromises one’s treatment he or she will definitely die. So, these rumours scare us.’ MC, IDI, male participant.

### Self-efficacy

The HCWs at NN reported that their experience integrating HIV/NCD care for more than three years promoted their knowledge and skills in managing NCDs and HIV confidently.

‘…. but then when you are coming to the clinic, you are always prepared to say, “I am going to the clinic and I will see a hypertension client, a diabetes client who also is on ART client” and you are always prepared for all these conditions. So, it is a good system and from my experience I have seen that it is really working well.” NN, FGD, female clinician.

‘I will use my own experience; I was afraid before coming in here to be able to manage diabetes in individuals with HIV. I wasn’t even sure on how to go about it, but through this integration, it boosts up my confidence because right now I know when to start giving the medication or not, or even adjust the medication on the patient, things like those because we have seen these things here. NN, FGD male clinician.

Table 4 is a summary of the facilitators and barriers to integrating screening of hypertension, DM and dyslipidaemia at the district ART clinics in the study.

### Results from the Observations

At NN, it was observed that within the clinic, there were functional blood pressure machines, a glucometer and an HBA1c machine. The clinic also had a special phlebotomy and a small lab within the vicinity of the clinic. NN also had a functional laboratory within the district hospital, separate from the ART clinic which were functional and able to conduct lipid analysis in addition to the blood glucose and HBA1c analysis.

On the other hand, while both CZ and MC had functional BP machines within the ART clinics, they had no glucometers nor HbA1c machines for DM screening. Nonetheless, both these facilities had a laboratory facility outside the clinic but within the hospital with the capacity to measure blood glucose. The laboratories also had biochemistry machines which were able to perform lipid analysis, but machines were non-functional due to lack of reagents.

## DISCUSSION

The present study explored the barriers and facilitators to integrating screening for hypertension, DM and dyslipidaemia among adult PLWH at district hospital ART clinics in Southern Malawi. The quantitative aspect revealed an unexpectedly low prevalence of screening for conditions on commencement of ART and in the previous year before the study.

Both PLWH and HCW perceived the integration to have a relative advantage in terms of time efficiency, providing an opportunity to integrate patient information and retention in care. Moreover, the intervention was regarded as feasible since the set-up would utilise the already existing space and structures. Furthermore, facilities which performed poorly on the implementation of the integration were willing to perform a trial of the intervention. There were success reports on the implementation of the intervention strongly linked with NGO-supported initiatives for integrated care in terms of human resources availability and training, screening and management tools, and medication for the NCDs of interest. Regarding the individual level, an important facilitator was that PLWH were optimistic about the initiative, and both the PLWH and HCW had some knowledge on the link between HIV infection and its therapy and the NCDs. Reported barriers to uptake of the intervention included perceived long patient waiting time, additional workload in the presence of an already strained workforce, the need for system reorganisation and the cost associated with it. At the inner setting level, barriers included a lack of prioritization of NCDs screening in government-only-funded facilities which was linked to lack of resources and an enabling environment for the integration without NGO support and lack of incentives for staff in terms of training for the screening and management of NCDs at the ART clinics in government-only funded facilities. At the individual level, belief that the integration would lead to increased workload for the HCW, lack of knowledge and the fear of screening and diagnosis without available medication for management amongst PLWH were important barriers.

HIV infection has transformed from a fatal infection into a manageable chronic condition due to advancements in ART [34]. From the quantitative results of the present study, it was evident that the proportion of individuals who were categorised as underweight, based on BMI at the initiation of ART, reduced statistically significantly at the index visit. On the other hand, our results also showed that at least 20% of the PLWH whose les were reviewed were either overweight or obese, and the proportion of individuals who were overweight also significantly increased at the index visit compared to the time of ART initiation. Importantly, overweight and obesity have been linked to ART use among PLWH [35, 36], and it is important to recognise that HIV infection is associated with an increased risk of ASCVDs [37]. PLWH have a higher likelihood of developing ASCVDs attributed to HIV-associated inflammation and immune activation and ART, which may influence dysmetabolism and atherosclerosis, hence increased cardiovascular risks [38]. In addition to these factors, PLWH have a high prevalence of traditional cardiovascular disease risk factors, including high blood pressure, dyslipidaemia and type 2 DM, which significantly elevate the risk of ASCVDs [39]. Therefore, these traditional risk factors must be screened and managed early to curb the risk of cardiovascular events and mortality among PLWH. In Malawi, it is predicted that up to 20,000 PLWH will suffer ASCVD-related deaths annually by 2025, due to undiagnosed and poorly managed hypertension, dyslipidaemia and DM [13]. This emphasises the need to aggressively screen for these traditional risk factors and devise better implementation strategies toward achieving early detection and management of these conditions. We discuss here the results from the study on the barriers and facilitators to the intervention based on the three domains of the CFIR framework used for the qualitative aspect of this study, and the observations made.

### The Intervention

Integrating the screening and management of NCDs among PLWH is an effective intervention that improves early identification, management, and overall health outcomes [40], however not implemented well in most ART clinics in Malawi, and per the results of this study. In this study, both PLWH and HCW perceived the intervention to have a relative advantage in terms of time efficiency, providing an opportunity to integrate patient information and retention in care. Our findings mirror other findings within Africa which indicated that HCW and PLWH believe that integration is more effective in saving time, reducing transport costs, and improving client retention and clinical outcomes [41, 26]. These positive attitudes and beliefs regarding the intervention are crucial facilitators as they determine the adoption, implementation, and sustainability of the evidence-based practice at the clinics [42, 43]. Integrating care for DM and hypertension with HIV care in Sub-Saharan Africa has shown that the intervention is overall clinically effective [40]. The intervention favours the quality of care in terms of comprehensiveness, continuity of care, and patient satisfaction which outweighs the wait-time implications [44–46]. Additionally, the integration of screening for NCDs within HIV care programs has been shown to improve the identification of undiagnosed NCDs among PLWH and reduce the duplication and fragmentation of services, hence increasing the efficiency in using limited resources use [46].

The finding that the intervention was regarded as feasible due to the utilization of existing space and structures is a positive development. Leveraging existing resources can minimize the need for extensive infrastructure changes or additional investments, making the integration more practical and cost-effective [47]. This suggests that the integration of screening services for hypertension, DM, and dyslipidaemia can be implemented without significant disruption to the healthcare system. The relative success observed in NN in the present study proves that it is possible to build on existing facilities to create a system for integrated care for NCD/HIV care, which is in keeping with previous findings [48]. Moreover, the willingness of facilities which performed poorly to participate in a trial of the intervention is promising. It demonstrates their recognition of the potential benefits of the integration and their openness to exploring ways to improve their implementation efforts.

However, as stated by some of the HCWs in the study, long waiting times for the PLWH and increased workload for HCWs associates are frequent and anticipated challenges associated with the intervention, especially for health facilities that have not yet implemented the integration. These observations are not unique to Malawi and have been shared from other African countries [46, 49–52]. Thus, there is a need to carefully consider implementation strategies for integration to minimise the workload and waiting times while maximising the gains associated with it. Task-shifting, defined as the rational movement of some care duties from physicians to non-physician healthcare workers, and task-sharing is a planned strategy in which a team of healthcare professionals work together to deliver a service [40], accompanied by training or certification and support for healthcare workers are some strategies which can curb the challenge of workload and long waiting times for clients while integrating the screening NCDs [53, 54]. Task-shifting and task-sharing have been demonstrated to be effective in screening hypertension for example, and have been recommended by the WHO for low and middle-income countries such as Malawi due to the low healthcare worker-to-patient ratio [55].

### The inner setting

Successful implementation in NN in the present study was associated with NGO-supported initiatives, which provided vital human resources, screening tools and training for NCD screening and management for the HCW. Additionally, medication availability for NCDs played a crucial role in facilitating the integration. These results confirm the feasibility of integrating screening and management for NCDs into HIV services in a Malawian setting, but that it also requires commitment in terms of resources. Resource allocation is crucial to ensure the availability of screening tools, diagnostic equipment, and expertise within the healthcare system. Incentives for staff, including training opportunities, can improve their engagement and competence in NCD screening and management [19, 47, 56]. These initiatives are attainable.

Recent studies have indicated that the intervention is financially feasible and associated with positive clinical outcomes, improved access to care, and successful implementation in rural Malawi [57]. Thus, the barriers aired in this study related to the lack of prioritisation and limited opportunities for continuing professional development or training for HCW may be addressed with adequate government commitment. The District Hospital ART facilities may also leverage NGO support and channel resources toward effective initiatives such as the integration of HIV/NCDs care, toward ameliorating the morbidity and mortality associated with cardiovascular diseases, which are the leading cause of mortality and higher among PLWH.

Furthermore, efforts should be made to strengthen coordination and collaboration between HIV and NCD care services so that the integrated approach may leverage the optimal use of resources such as available HCW, rendering the intervention cost-effective [56]. Facilities may remodel the delivery of care, including the development of integrated care protocols and guidelines and the reorganisation of patient ow to ensure context adaptability of the intervention to promote a successful implementation of the integration [46, 50].

### The individual level

At the individual level, one significant facilitator was the optimism exhibited by PLWH about the initiative, coupled with the confidence of the HCW to conduct the screening. This positive outlook can be leveraged to increase acceptance and participation in the screening process [58] by the PLWH as the recipients and primary beneficiaries of the initiative and the HCW as the providers. Additionally, the presence of some knowledge among both PLWH and HCWs regarding the association between HIV infection, ART and cardiometabolic derangements can play a crucial role in promoting a better understanding and uptake of the screening [58–59]. However, barriers such as the belief among HCWs that the integration of screening for NCDs would result in an increased workload for them need addressing. The task-shifting and task-sharing discussed in this study may be leveraged to render the initiative successful, cost-effective and HCW-friendly in rural settings [55, 57]. The expression by PLWH on the fear of screening and diagnosis without readily available medication for managing the identified conditions cannot be ignored. The availability of medication for management and screening equipment is an essential facilitator to integrating cardiometabolic care in HIV-related services in low-income settings [60]. Thus, by providing adequate support to HCW and ensuring that necessary knowledge and resources are available, this integration’s success and potential benefits can be maximised in Malawi.

### Availability of screening and diagnostic equipment

The importance of having functional diagnostic or screening equipment for the successful integration of screening of hypertension, dyslipidaemia and DM within the ART clinic cannot be overstated. From the results of the present study, it is evident that the availability of functional diagnostic and screening equipment at NN, led by a non-governmental organisation initiative, significantly influenced its performance in successfully implementing the integration intervention. On the contrary, CZ and MC, which were low-performing facilities and government-led, had unavailable biochemistry equipment for DM or lipid analysis equipment within the clinic vicinity and often nonfunctioning blood pressure machines. These results are also shared by other low-income settings, where lack of functional diagnostic tools poses a barrier to the intervention [19, 21, 46, 61].

To improve the screening and management of hypertension, DM and dyslipidaemia in such low-income settings, the availability of functional blood pressure machines is crucial [19]. And, in settings where task-shifting and/or sharing is involved, automated blood pressure cuffs may be preferable as they eliminate the need for expertise while ensuring accuracy in low-income settings [62]. Where the availability of standard laboratory within the clinic is a challenge, point-of-care portable devices for analysing blood glucose and a lipogram can play a vital role in providing these essential services. These devices, such as a portable HBA1C machine, glucometer and portable lipogram machine, are relatively inexpensive, easy to use, and can provide results in a matter of minutes, making them clinically effective, cost-effective and hence ideal for use in low-income settings [63]. Additionally, for DM screening, the point of care HBA1C measurements can indicate individuals in the pre-diabetes category for early management [64]. Point-of-care portable devices can, therefore, play a valuable role in integrating screening and management of hypertension, dyslipidaemia, and DM in low-income settings. Still, their use in a setting such as Malawi district ART clinics among PLWH is yet to be evaluated.

## LIMITATIONS

This study has some limitations that should be considered when interpreting its findings. Firstly, it only assessed three of the five domains of the CFIR framework, focusing on the intervention, inner setting, and individual aspects while omitting the assessment of the outer setting and the process. This limited scope may not provide a comprehensive understanding of the complete contextual factors influencing the integration of screening for hypertension, DM, and dyslipidaemia among adult people living with HIV in the district hospital ART clinics of Southern Malawi. Additionally, as the study was observational in nature, it is important to note that it cannot establish causation or determine cause-and-effect relationships. While the mixed-method approach, incorporating both quantitative and qualitative aspects, strengthens the study’s evidence base on the barriers and facilitators to integration, these limitations should be considered when interpreting the study’s conclusions.

## CONCLUSION

The study findings revealed poor integration of screening of hypertension, DM and dyslipidaemia among PLWH in Southern District Hospital ART clinics in Malawi, highlighting a significant gap in care. Nonetheless, the integration was perceived to have advantages, including time efficiency, cost-effectiveness and the potential for patient information integration and retention in care. Barriers such as increased workload for HCW were acknowledged, requiring strategic implementation strategies like task-shifting and task-sharing. The importance of resource allocation and the coordination between HIV and NCD care services were underscored. The study emphasizes the feasibility of the intervention due to its potential to utilize existing resources and structures. It highlights the critical role of the availability of sustainable screening and diagnostic equipment such as point-of-care portable devices, to address barriers to screening and managing NCDs effectively in low-income settings such as Malawi.

## Figures and Tables

**Table 1:** Sampling technique and sample size for the qualitative aspect of the study

Participant type	Method of Data collection	Number				Sampling technique	Rationale
		NN	CZ	MC	Total		
PLWH	In-depth Interviews	8	10	8	26	Purposive	These are beneficiaries of the services
ART clinic staff	Focus group discussions	1	1	1	3	Purposive	These are health providers for the services
DHMT Members	Key informant interviews	1	1	1	3	Purposive	These are decision makers for the district hospitals

Key: NN = Neno, CZ = Chiradzulu, MC = Mangochi, District ART clinic facilities; PLWH = People living with HIV; DHMT = District Health Management Team.

**Table 2:** CFIR domains and constructs explained

CFIR Domain	CFIR construct	Explanation (facilitator or barrier)
**Intervention**	Intervention Source	Perception on involvement of healthcare providers on the intervention
	Evidence strength and quality	Perceived strength of evidence motivating the implementation of integration of NCD screening in ART clinics
	Relative advantage	Perceived benefits of screening NCDs in PLWH at the ART clinic
	Adaptability	Ideas on how best the ART clinic can accommodate the integration of screening for NCDs
	Trialability	Willingness to pilot the integration of screening for NCDs at the ART clinic if not yet implemented
	Complexity	Perceived difficulties with implementing the integration of screening for NCDs in PLWH at the ART clinic
	Cost	Views on cost implications of integrating the screening of NCDs in PLWH at the ART clinic
**Inner Setting**	**Implementation Climate**	
	Compatibility	Whether integration of screening for the conditions would be compatible within existing workflow of the clinic
	Organisational Incentives and rewards	Whether availability/lack of functional machines and medicines for the conditions facilitate or hinder the integration
	**Readiness for Implementation**	
	Available resources	Availability/lack of diagnostic gadgets, human resource
	Access to knowledge and Information	Awareness by PLWH screening and management of hypertension, DM and dyslipidaemia at ART clinic. Training of Healthcare providers on screening for hypertension, dyslipidaemia and DM
**Characteristics of Individuals**	Knowledge and beliefs about the intervention	Knowledge and beliefs for the screening for hypertension, DM and dyslipidaemia by healthcare workers and PLWH
	Self-Efficacy	Confidence of healthcare providers in their own ability to screen and prescribe medicines for hypertension, DM and dyslipidaemia in ART clinics

**Table 2:** Summary of participant characteristics

Variable	Statistic		Significance (where applicable)
**Sex (Male)**	55%		
**Age**			
Median (IQR)	49 (36–56)		
**Duration on ART**			
Median months (IQR)	41 (20–57)		
**ART Regimen**	**Baseline**	**At time of study**	
tenofovir/lamivudine/efavirenz	59%	1%	
tenofovir/lamivudine/dolutegravir	40%	98%	
Other lines	1%	1%	
**BMI**	**Baseline**	**At time of study**	**P-value**
Median (IQR)	21.1 (19.1–23.4)	21.9 (19.7–24.5)	<0.001
Underweight	18.7%	13.7%	0.002
Normal	66.2%	65.0%	0.30
Overweight	10.7%	15.6%	0.001
Obese	4.4%	5.4%	0.17
**Occupation**			
Formal (office)	16.6%		
Casual labour	21.0%		
Other (small scale businesses)	21.0%		
Housewife	9.8%		
Unrecorded	31.5%		

Overall, 8%, 1.8% and 0% were screened for hypertension, type 2 DM and dyslipidaemia, respectively, at the commencement of ART as shown in the chart below. Similarly, within the previous 12 months from the study time, only 7.9%, 1% and none were screened for hypertension, type 2 DM and dyslipidaemia respectively.

Only one district hospital ART clinic facility (NN) virtually performed the screening for hypertension and DM. It recorded 40% and 9.84% for hypertension and DM respectively at the commencement of ART and recorded 39.4% and 5.14% screening for hypertension and DM within the previous year.

**Table 3:** shows the summary characteristics of participants who took part in the qualitative aspect of the study.

	IDI participants (n=26)	FGDs participants (n=21)	KII participants (n=3)
**Age (Median, IQR)**	50 (47 – 560)	37 (35 – 41)	40 (29 – 41)
**Sex (%)**			
Female	71	48	33
**Education Level (%)**			
None	13	0	0
Primary	54	0	0
Secondary	33	19	0
Tertiary	0	81	100
**Presence of NCDs (%)**			
Hypertension	29	N/A	N/A
DM	4	N/A	N/A

**Table 4:** Facilitators and barriers to screening for NCDs at the ART clinics in the study

CFIR domain	Facilitators	Barriers
**1. Intervention** ***Relative advantage*** ***Complexity, trialability, and cost***	Time efficient Integrated patient information Opportunity for better client retention Use of already existing set-up Perceived cost-effectiveness Willingness for trial	Long patient waiting time Additional workload Infrastructure challenges and need for system reorganization Expensive system to set up
**2. Inner setting** ***Readiness for implementation*** ***Organisational incentives and access to training***	Success stories in NGO-supported initiatives for integrated care Partnerships with NGOs facilitate continuing professional development	Lack of prioritization in government only-funded facilities linked to lack of resources without NGO support Limited opportunities for continuing professional development/training funded by the government
**3. Characteristics of the individual** **Knowledge and beliefs** **Self-efficacy**	PLWH optimistic about the initiative Knowledge on HIV-NCDs link HCWs confidence to screen and manage NCDs	Perceived increased workload for HCWs Lack or poor knowledge on HIV-NCDs link Fear of screening and diagnosis with lack of medication for management amongst PLWH

## Data Availability

The datasets used and/or analysed during the current study are available from the primary author upon reasonable request.

## Abbreviations

AIDS: Acquired immune deficiency disease syndrome
ART: antiretroviral therapy
ASCD: atherosclerotic cardiovascular disease
CFIR: consolidated framework for implementation research
COMREC: College of medicine research and ethics committee
DM: Diabetes Mellitus
HIV: Human immune deficiency virus
KUHes: Kamuzu university of Health sciences
MHIRST: Malawi HIV Implementation Research Training Program
NCD: Non-Communicable diseases
PLWH: people living with HIV
